# Arthritis prevention in the pre-clinical phase of RA with abatacept (the APIPPRA study): a multi-centre, randomised, double-blind, parallel-group, placebo-controlled clinical trial protocol

**DOI:** 10.1186/s13063-019-3403-7

**Published:** 2019-07-15

**Authors:** Mariam Al-Laith, Marianna Jasenecova, Sonya Abraham, Aisla Bosworth, Ian N. Bruce, Christopher D. Buckley, Coziana Ciurtin, Maria-Antonietta D’Agostino, Paul Emery, Hill Gaston, John D. Isaacs, Andrew Filer, Benjamin A. Fisher, Thomas W. J. Huizinga, Pauline Ho, Clare Jacklin, Heidi Lempp, Iain B. McInnes, Arthur G. Pratt, Andrew Östor, Karim Raza, Peter C. Taylor, Dirkjan van Schaardenburg, Dharshene Shivapatham, Alison J. Wright, Joana C. Vasconcelos, Joanna Kelly, Caroline Murphy, A. Toby Prevost, Andrew P. Cope

**Affiliations:** 1 Centre for Rheumatic Diseases, Department of Inflammation Biology, School of Immunology and Microbial Sciences, Faculty of Life Sciences and Medicine, King’s College London, Weston Education Centre, 10 Cutcombe Road, London, SE5 9RJ UK; 2 Department of Rheumatology, National Institute for Health Research-Wellcome Clinical Research Facility, Hammersmith Hospital, Imperial College, London, W12 0HS UK; 3 National RA Society, The Switchback Office Park, Gardner Road, Maidenhead, SL6 7RJ UK; 4 Arthritis Research UK Centre for Epidemiology, Centre for Musculoskeletal Research, Faculty of Biology, Medicine and Health, Stopford Building, University of Manchester, Oxford Road, Manchester, M13 9PT UK; 5 National Institute for Health Research Biomedical Research Centre and the Kellgren Centre for Rheumatology, Manchester University NHS Foundation Trust, Manchester Academic Health Science Centre, Manchester, M13 9WL UK; 6 Rheumatology Research Group, Institute of Inflammation and Ageing, College of Medical and Dental Sciences, University of Birmingham, Queen Elizabeth Hospital, Birmingham, B15 2WB UK; 7 Sandwell and West Birmingham Hospitals NHS Trust, West Bromwich, West Midlands, B71 4HJ UK; 8 Department of Adolescent and Adult Rheumatology, University College London Hospitals NHS Trust, 3rd Floor Central, 250 Euston Road, London, NW1 2PG UK; 9 Rheumatology Department, Hôpital Ambroise Paré, 92100 Boulogne-Billancourt, France; 10 INSERM U1173, Laboratoire d’Excellence INFLAMEX, UFR Simone Veil, Versailles-Saint-Quentin University, 78180 Saint-Quentin en Yvelines, France; 11 Section of Musculoskeletal Disease, Leeds Institute of Molecular Medicine, University of Leeds, UK NIHR Biomedical Research Unit, Leeds Teaching Hospitals NHS Trust, Leeds, LS4 7SA UK; 12 Department of Medicine, University of Cambridge and Addenbrookes Hospital NHS Trust, Cambridge, UK; 13 Musculoskeletal Research Group, Institute of Cellular Medicine, Newcastle University, 3rd Floor William Leech Building, The Medical School, Framlington Place, Newcastle upon Tyne, NE2 4HH UK; 14 Newcastle upon Tyne Hospitals NHS Foundation Trust, Newcastle upon Tyne, NE7 7DN UK; 15 Department of Rheumatology, Leiden University Medical Centre, Leiden, The Netherlands; 16 Institute of Infection, Immunity and Inflammation, College of Medical, Veterinary and Life Sciences, University of Glasgow, 120 University Place, Glasgow, G12 8TA UK; 17 Botnar Research Centre, Nuffield Department of Orthopaedics, Rheumatology and Musculoskeletal Sciences, University of Oxford, Windmill Road, Headington, Oxford, OX3 7LD UK; 18 Amsterdam Rheumatology and immunology Center, locations Reade and Amsterdam University Medical Center, Amsterdam, The Netherlands; 19 Clinical, Education & Health Psychology Division of Psychology & Language Sciences, Faculty of Brain Sciences, University College London, London, WC1E 6BT UK; 20 Imperial Clinical Trials Unit, School of Public Health, Imperial College London, Stadium House, 68 Wood Lane, London, W12 7RH UK; 21 King’s Clinical Trials Unit, King’s College London, Institute of Psychiatry, 16 De Crespigny Park, London, SE5 8AF UK

**Keywords:** Rheumatoid arthritis, At risk, Pre-clinical phase, Intervention, Abatacept, Double-blind, Antibodies to citrullinated protein antigens, Autoantibodies, Placebo-controlled, Randomised

## Abstract

**Trial design:**

We present a study protocol for a multi-centre, randomised, double-blind, parallel-group, placebo-controlled trial that seeks to test the feasibility, acceptability and effectiveness of a 52-week period of treatment with the first-in-class co-stimulatory blocker abatacept for preventing or delaying the onset of inflammatory arthritis.

**Methods:**

The study aimed to recruit 206 male or female subjects from the secondary care hospital setting across the UK and the Netherlands. Participants who were at least 18 years old, who reported inflammatory sounding joint pain (clinically suspicious arthralgia) and who were found to be positive for serum autoantibodies associated with rheumatoid arthritis (RA) were eligible for enrolment. All study subjects were randomly assigned to receive weekly injections of investigational medicinal product, either abatacept or placebo treatment over the course of a 52-week period. Participants were followed up for a further 52 weeks. The primary endpoint was defined as the time to development of at least three swollen joints or to the fulfilment of the 2010 American College of Rheumatology/European League Against Rheumatism (ACR/EULAR) classification criteria for RA using swollen but not tender joints, whichever endpoint was met first. In either case, swollen joints were confirmed by ultrasonography. Participants, care givers, and those assessing the outcomes were all blinded to group assignment. Clinical assessors and ultrasonographers were also blinded to each other’s assessments for the duration of the study.

**Conclusions:**

There is limited experience of the design and implementation of trials for the prevention of inflammatory joint diseases. We discuss the rationale behind choice and duration of treatment and the challenges associated with defining the “at risk” state and offer pragmatic solutions in the protocol to enrolling subjects at risk of RA.

**Trial registration:**

Current Controlled Trials, ID: ISRCTN46017566. Registered on 4 July 2014.

**Electronic supplementary material:**

The online version of this article (10.1186/s13063-019-3403-7) contains supplementary material, which is available to authorized users.

## Background

Rheumatoid arthritis (RA) is a common chronic inflammatory immune-mediated disease of joints afflicting more than 500,000 subjects in the UK [1, 2]. If not adequately treated, the condition leads to destruction of synovial joints and significant disability. RA is costly to individuals and their families; one third of patients with arthritis stop work within 2 years of onset because of the deterioration in quality of life associated with their disease [3]. In the UK, RA is costly to the economy; the cost is estimated to be in the region of £5 billion per year through direct costs to the National Health Service (NHS) and associated healthcare providers and indirect costs associated with early mortality and loss of productivity [4].

The introduction of therapeutic strategies that focus on early, intensive treatment with conventional synthetic and biological disease-modifying anti-rheumatic drugs (DMARDs) has transformed the treatment of RA. Clinical trials have demonstrated that this approach leads to higher proportions of patients achieving periods of sustained clinical remission. This is associated with improved function and slowing or even prevention of joint damage over time. Indeed, intensive “treat-to-target” strategies in patients with very early RA can lead to extended periods of drug-free remission in a subset of patients [5]. If the pre-clinical phase of disease could be defined with accuracy, targeting therapy to those at highest risk of developing disease would have the potential to prevent or at the very least delay the onset of RA. Were such an approach to be successful, the disabling symptoms and signs of arthritis and the potential risks of unemployment could be prevented. Furthermore, the development of potentially life-threatening co-morbidities associated with chronic inflammatory diseases, such as cardiovascular disease and infection, could be substantially reduced.

The combination of serum antibodies to citrullinated protein antigens (ACPAs) and joint pain (termed arthralgia), in the absence of clinically detectable synovitis, is considered to most accurately define subjects at high risk of progressing to RA [6–8]. Data from longitudinal observational cohorts indicate that about 40% of high-risk subjects progress to clinically apparent arthritis within 18 months [9]. The combination of IgM rheumatoid factor (RF) and high serum ACPA levels defines those at highest risk; recent unpublished data indicate that the risk of developing disease in subjects with high titre ACPA in the absence of RF may be comparable to that in ACPA^+^RF^+^ subjects [9]. It follows from this that ACPA^+^RF^+^ or ACPA^+^RF^−^ arthralgia individuals would provide a valid target population for therapeutic intervention aimed at prevention of the syndrome we recognise as RA.

Abatacept is a fusion protein composed of the Fc region of the immunoglobulin IgG1 fused to the extracellular domain of cytotoxic T-lymphocyte antigen 4 (CTLA-4) [10]. Specifically, abatacept, a first-in-class co-stimulatory blocker, is a biologic DMARD that targets immune reactions early in the chain of events leading to inflammation in RA. It functions by interrupting the interaction between T cells and antigen-presenting cells, attenuating the co-stimulatory signals required for T-cell activation, differentiation and effector responses [11]. This results in downstream immunomodulatory effects on other inflammatory cells of the immune system.

In patients with established RA, administration of abatacept is associated with statistically significant and clinically meaningful improvements in the signs and symptoms, physical function and health-related quality of life. It inhibits structural damage in a broad spectrum of patients [12, 13], including those with early or established disease [14] and those with an inadequate response to tumour necrosis factor (TNF) blockade [15]. Interestingly, disease activity scores (DASs) have been shown to continue to improve beyond 12 months, indicating that the beneficial effects of co-stimulatory blockade not only are sustained but accumulate over extended periods of time.

In the ADJUST study, abatacept demonstrated good efficacy in patients with very early RA, including ACPA^+^ patients with undifferentiated arthritis, delaying onset of RA when compared with placebo, and promoting sustained remission rates and reduced radiographic progression, even after therapy is withdrawn [16]. More recent phase III studies have confirmed the efficacy of weekly subcutaneous (SC) injections of abatacept [17]. Clinical efficacy as measured by American College of Rheumatology 20 (ACR 20) and Health Assessment Questionnaire (HAQ) response was similar between SC abatacept and intravenous (IV) abatacept. Clinical trials and post-marketing surveillance suggest that the drug has an acceptable safety profile, particularly with respect to infection.

Although abatacept has a very favourable safety profile, suggesting that this approach might be acceptable to individuals during the pre-clinical phase of RA, there were no published studies at the time of APIPPRA (Arthritis Prevention In the Pre-clinical Phase of RA with Abatacept) study protocol development investigating the acceptability of targeted treatment strategies during the pre-clinical phase of RA. Therefore, understanding the factors that influence the acceptability of this therapeutic approach and the willingness of at-risk subjects to participate in interventional studies will be fundamental to trial methodology and the success of preventative, personalised medicine in the future.

## Methods/Design

### Study objectives

The principal objective of the study is to determine whether RA can be prevented or delayed if targeted immunotherapy is administered to subjects in whom autoantibody screening indicates a high risk of developing disease. Specifically, this study will test the hypothesis that the onset of arthritis in an “at risk” group can be prevented or delayed by weekly injections of abatacept for a period of 52 weeks.

The specific aims of study are to do the following:

1. Evaluate the feasibility, efficacy and acceptability of abatacept therapy in subjects at high risk of developing RA and
2. Characterise immune and inflammatory responses associated with ACPA before, during and after therapy with abatacept.

### Trial design

The APIPPRA trial is a randomised, placebo-controlled, double-blind multi-centre trial undertaken at 31 specialist rheumatology clinics across the UK and the Netherlands. The trial was designed to ascertain the effectiveness of abatacept in ACPA^+^RF^+^ or ACPA^high^RF^−^ subjects with arthralgia who are deemed to be at high risk of developing RA. Eligible subjects will be randomly allocated to receive weekly injections of either abatacept or placebo treatment over the course of a 52-week period. This provides the best chance of establishing whether differences observed between the two groups are due to the treatment.

**Fig. 1:**
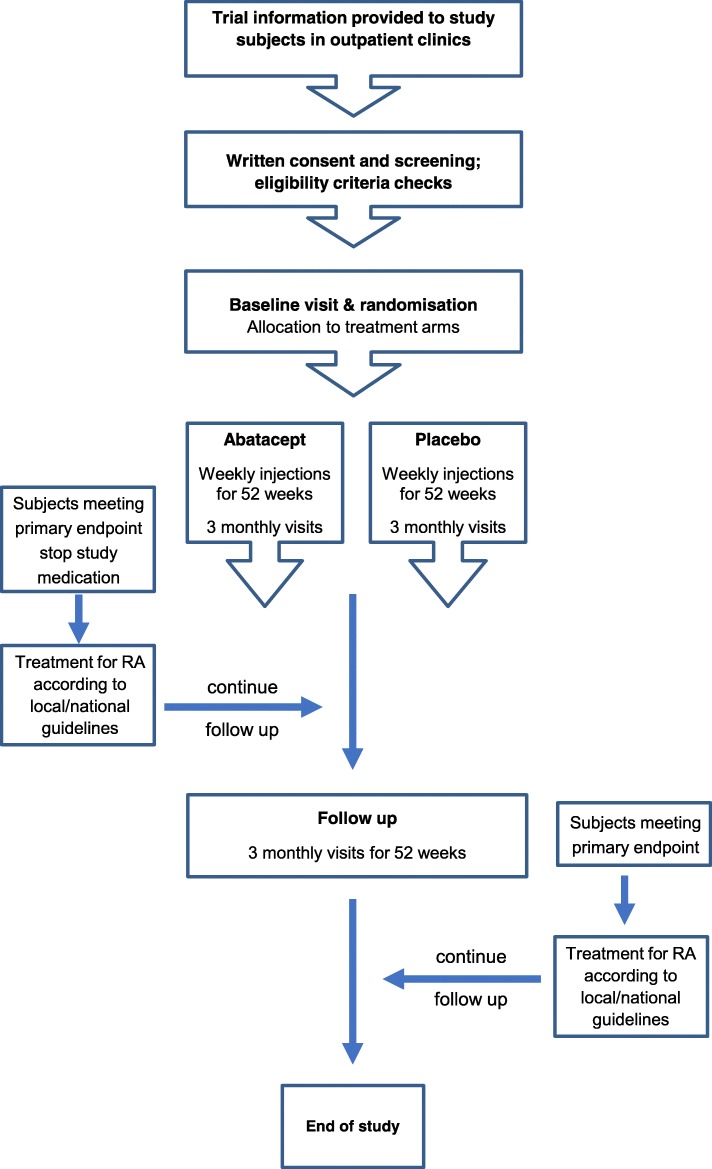
Study design and flow chart

The SPIRIT checklist is included as (Additional file 4). The study design is shown in Fig. 1.

### Study population

The APIPPRA study will recruit study subjects who are at high risk of developing RA and who show no clinical evidence of joint swelling. Participants may be recruited by using a range of ethically approved methods, including during rheumatology out-patient clinics, following referral to recruiting centres from other rheumatology out-patient clinics, referrals from general practitioner (GP) practices through the routine referral pathway or patient identification centre (PIC) pathway, or through existing research databases and registries. Subjects may be identified by pre-screening of laboratory results for RF and ACPA.

### Eligibility criteria

#### Inclusion criteria

1. Male or female subjects who are at least 18 years old.
2. Arthralgia, defined as non-traumatic joint pain localised to synovial joints, including (but not necessarily confined to) hands, wrists or feet and considered (by the supervising rheumatologist) to be inflammatory in nature.
3. Positive for serum RF and ACPA as defined by local clinical laboratory testing. Subjects who are RF-negative but who carry high levels of serum ACPA (defined as being at least 3 × upper limit of normal [ULN] for the assay) may be included.
4. Able and willing to give written informed consent and comply with the requirements of the study protocol (Additional file 1).

#### Exclusion criteria

Target disease exceptions:

1. Previous diagnosis of RA or other form of inflammatory arthritis, including (but not limited to) systemic lupus erythematosus, psoriatic arthritis, ankylosing spondylitis, gout or pyrophosphate arthropathy and including current treatment with DMARDs or biological therapy.
2. Arthralgia that, in the opinion of the supervising physician, is poorly localised (e.g., pelvic or shoulder girdle pain, pain that is confined to the axial skeleton or entheses, or pain which the physician considers to be due to osteoarthritis or fibromyalgia or related to other autoimmune conditions such as type I diabetes, coeliac or autoimmune thyroid disease).
3. Clinically apparent inflammatory arthritis, as assessed by a rheumatologist, characterised by soft tissue swelling of one or more synovial joints. Subclinical synovitis, as detected by imaging modalities such as ultrasonography or magnetic resonance imaging, is not an exclusion criterion.
4. A history of oral or parenteral use of corticosteroids within the last 12 weeks used to treat the current episode of musculoskeletal symptoms.
5. Co-morbidities requiring chronic treatment with immunosuppressive or immune modulating therapy.
6. Subjects who have at any time received treatment with any investigational drug within 28 days of the first dose of study drug.
7. A history of acute allergic reactions to biological therapy or immunoglobulins.

Medical history and concurrent diseases:

1. Subjects who are incapable of completing study-related assessments or give informed consent (see Additional file 1).
2. Subjects with current symptoms of severe, progressive or uncontrolled renal, hepatic, hematologic, gastrointestinal, pulmonary, cardiac, neurologic or cerebral disease, whether or not related to RA and which, in the opinion of the investigator, might place a subject at unacceptable risk for participation in the study.
3. Subjects with a history of cancer, other than non-melanoma skin cell cancers cured by local resection or carcinoma *in situ*, in the last 5 years.
4. Subjects with tuberculosis (TB), including those at high risk of TB, chronic viral infections or recent serious bacterial infections; subjects receiving live vaccinations within 3 months of the anticipated first dose of study medication; or those with chronic illnesses that would, in the opinion of the investigator, put the participant at risk.
5. Subjects who currently abuse drugs or alcohol.
6. Subjects who are pregnant or breastfeeding or women of child-bearing potential not willing to use adequate contraception during the period of investigational medicinal product (IMP) dosing and for up to 14 weeks after the last dose of study drug.
7. Male subjects not willing to use adequate contraception during the period of IMP dosing and for up to 14 weeks after the last dose of study drug.

##### Physical and laboratory tests at screening

Subjects must not test positive for the following:

1. Latent tuberculous infection according to the interferon gamma release assay. Subjects who are positive and who have received treatment for at least 4 weeks may be selected.
2. Antibodies to hepatitis B surface antigen.
3. Hepatitis C antibody if the presence of hepatitis C virus was also shown with polymerase chain reaction or recombinant immunoblot assay.
4. HIV.

Subjects with any of the following laboratory values or other test results that, in the opinion of the principal investigator, might place a subject at unacceptable risk for participation in the study:

1. Haemoglobin < 85 g/L
2. White blood cell count < 3 × 10^9^/L
3. Platelets < 100 × 10^9^/L
4. Serum creatinine > 2 times the ULN
5. Serum alanine transaminase (ALT) or aspartate aminotransferase (AST) > 2 times the ULN.

### Trial intervention

#### Investigational medicinal product/placebo schedule and administration

Abatacept will be administered by SC injection at a dose of 125 mg per injection (125 mg/mL). Placebo is supplied as identically labelled injections containing a sterile saline solution for SC administration. IMP is supplied as kits of four pre-filled syringes with coded label. Given that there are no guidelines or therapies licensed for treating subjects at risk of RA and that close monitoring of signs and symptoms until the development of swollen joints is standard of care, the use of placebo was deemed to be acceptable in this clinical trial.

#### Treatment period (weeks 0–52)

Following randomisation, participants will start their dosing regimen at the baseline visit. Abatacept (or placebo) will be injected weekly at the recommended dosage of 125 mg/mL for 52 weeks. Participants are trained to self-administer study drug subcutaneously (for the first injection) using the single-dose pre-filled syringe in accordance with local practices for the administration of biological therapy as part of standard care. Participants will be given 3 months’ worth of study medication at baseline and at each subsequent 3 monthly scheduled visits during the first 12 months. Participants will also be given information about the study drug, such as the Arthritis Research UK (now Versus Arthritis) Drug Information leaflet, in accordance with local practise. Subjects will also be given a study medication diary card to record their weekly injections.

In the event of missed doses, participants should not take their medication unless it is within ± 3 days of the scheduled medication dosing date. In addition to the 3 monthly visits, there will be brief telephone consultations, once a month, to check that study subjects are administering their weekly injections and to ask whether there have been any changes in their symptoms.

#### Follow-up period (weeks 53–104)

Once participants have completed the 52-week course of IMP/placebo, they will be seen in the outpatient clinic every 3 months for assessments (see Table 1; Schedule of blood and urine sampling) similar to those in year 1 in order to monitor the impact of the treatment phase. This follow-up period is especially important because, if at any time participants develop new joint pains or swelling, they will be assessed promptly and treated appropriately in a similar way that clinical staff would assess any new patient presenting with similar symptoms.

**Table 1:** Trial Flowchart and Schedule of Visits

Assessment		Study treatment				Follow up				
Visit No.^a^	1	2	3	4	5	6	7	8	9	10
WEEKS	Screening	Baseline	13	26	39	52	65	78	91	104
Registration Form	X									
Demographics	X									
Eligibility checks ^b^	X									
Medical History	X									
Physical Examination		X		X		X		X		X
Disease Activity Assessments		X	X	X	X	X	X	X	X	X
X-rays of hands & feet ^c^	X^c^									
Ultrasonography of symptomatic joints and limited joint set ^d^		X^d^		X^d^		X^d^		X^d^		X^d^
ACR/EULAR remission		X	X	X	X	X	X	X	X	X
IMP supply for weekly SC dosing ^e^		X	X	X	X					
Treatment Log (IMP)			X	X	X	X				
ESR & CRP ^f^	X		X	X	X	X	X	X	X	X
Routine Bloods (Haem & Biochem) ^g n^	X^g^		X	X	X	X	X^n^	X^n^	X^n^	X^n^
Screening Bloods (HIV, Hep B& C, TB) ^c^	X^c^									
Chest X-ray ^c^	X^c^									
Lifestyle Factors Questionnaire		X		X		X		X		X
Symptoms in Persons At Risk of Rheumatoid Arthritis (SPARRA) questionnaire ^h^		X^h^				X^h^				
Health Assessment Questionnaire (HAQ)		X	X	X	X	X	X	X	X	X
Modified Illness Perception Questionnaire (IPQ-R)		X		X		X		X		X
Euro-Quality of Life Questionnaire (EQ-5D)		X		X		X		X		X
Hospital Anxiety and Depression Scale (HADs)		X		X		X				X
Functional Assessment Of Chronic Illness Therapy-Fatigue Questionnaire (FACIT-F)		X		X		X		X		X
RA Work Instability Scale (RA-WIS)		X		X		X		X		X
Perceptions of Trial Participation Questionnaire ^i^	X^i^									
Concomitant Medication		X	X	X	X	X	X	X	X	X
Adverse Events		X	X	X	X	X	X	X	X	X
Compliance checks ^j^			X	X	X	X				
Status Form			X	X	X	X	X	X	X	X
Withdrawal Form ^k^			X^k^	X^k^	X^k^	X^k^	X^k^	X^k^	X^k^	X^k^
Blood/Urine for biomarkers ^l^		X	X	X	X	X	X	X	X	X

^a^Flexibility of scheduled visits will be allowed of +/- 2 weeks either side of visit

^b^Eligibility criteria

^c^These tests can be used if taken up to 12 weeks before screening

^d^Ultrasonography must be done before treatment is initiated at baseline visit and within 2 weeks either side of the scheduled 6, 12, 18 and 24 month Visit

^e^Three months worth of IMP will be supplied for weekly subcutaneous dosing. Missed doses are only allowed if within ± 3 day window

^f^These assessments will not be necessary if the baseline visit is scheduled within two weeks of the screening visit

^g^These tests may be undertaken as part of routine assessments

^h^SPARRA questionnaire will be given to participants to be completed in their own time after the study visit, to be returned to the local study team by post

^i^Potential participants, including those that do not wish to participate in the APIPPRA study, will complete the Perceptions of Trial Participation Questionnaire following written consent

^j^Monthly telephone calls will also be used to address compliance during IMP treatment period

^k^As required

^l^See Table 2, for schedule of sampling for exploratory biomarkers

^m^Subjects will be randomised following baseline clinical assessments, ultrasonography and blood/urine samples

^n^Routine bloods beyond the 12 month study visit will be taken only if clinically indicated, and left to the discretion of the supervising physician

#### Concomitant medication

For the duration of the trial, the investigator or another healthcare professional (for example, GP) may prescribe simple analgesics or non-steroidal anti-inflammatory drugs considered necessary for the treatment of the participant’s joint symptoms. Treatments for concurrent non-rheumatic disorders will be given as needed provided that they are not expected to interfere with the evaluation of the study medication. However, the following drugs are not permitted before the onset of clinically apparent synovitis (primary endpoint):

- Oral or parenteral glucocorticoids. Short courses (<2 weeks) of oral or parenteral steroids will be permitted for the treatment of significant, non-rheumatic, concurrent illnesses, including but not confined to asthma and chronic obstructive pulmonary disease.
- DMARDs.
- Any other biological agent for the treatment of RA.
- Any other medicinal product that, in the supervising physician’s opinion, may influence underlying disease activity through effects on immune or inflammatory responses or both (with the exception of non-steroidal anti-inflammatory drugs).

#### Treatment for study subjects reaching primary endpoint

For those study subjects achieving the primary endpoint (i.e., the development of clinically apparent synovitis or RA), the use of corticosteroids and DMARDs will be permitted, and the choice of therapy will be left to the discretion of the supervising physician. All subjects remain in the study and complete follow-up assessments in accordance with the schedule of visits (see Table 1, Trial flow chart), including full documentation of treatment for their inflammatory arthritis.

### Treatment-stopping rules

The trial may be prematurely discontinued by the sponsor or chief investigator (CI) on the basis of new safety information or recommendations given by the data monitoring and ethics committee to the trial steering committee. If the trial is prematurely discontinued, active participants will be informed, final data will be collected, and no further participant data will be collected thereafter. The research ethics committee will be informed within 15 days of the early termination of the trial.

### Study assessments

After screening, all participants will undergo baseline study visits and then 3 monthly follow-up visits for the duration of the trial. Clinical assessors will be blinded to joint assessments by ultrasonography.

Participants will follow the visit schedule summarised in Table 1 unless participants consider that they are experiencing a worsening of symptoms or have developed swelling of joints, in which case participants will be seen within 2 weeks. In addition to attending the 3 monthly assessments, they will be telephoned by their research nurse or designated staff involved in the study every month (during the treatment period) to check that study subjects are adhering to their study medication and managing their symptoms. Details of assessments and flexibility at scheduled visits are shown in Table 1.

The baseline assessment should be performed no later than four weeks after the screening assessment. For all subsequent assessments, if participants cannot attend on the due date, a two-week window either side of the assessment due date will be permitted.

#### Unscheduled visit assessments

These assessments will be undertaken for subjects experiencing worsening of symptoms or swelling of joints between scheduled visits. Study subjects will be seen promptly and usually within two weeks of new signs and symptoms developing.

1. Physical examination
2. Disease activity (includes extended joint counts 68/66, DAS28, simple disease activity index (SDAI), clinical disease activity index (CDAI), pain visual analogue scale (VAS) and physician and patient global assessments (VAS))
3. ACR/European League Against Rheumatism (ACR/EULAR) remission (as defined in [18])
4. Acute-phase reactants: erythrocyte sedimentation rate (ESR) and C-reactive protein
5. Patient questionnaires as listed above at baseline visit
6. Confirmation of clinically apparent synovitis by ultrasonography
7. Blood for biomarkers (this will replace the subsequent scheduled blood draw when the date of the unscheduled visit precedes the next scheduled visit by not more than four weeks)
8. Urine for biomarkers (this will replace the subsequent scheduled urine collection when the date of the unscheduled visit precedes the next scheduled visit by not more than four weeks).

#### Ultrasonography

Participants will also undergo imaging by ultrasonography. This part of the APIPPRA study will be undertaken in those recruiting centres that provide this service as part of routine clinical care and/or where there are personnel trained in musculoskeletal ultrasound using imaging equipment approved by the APIPPRA study investigators (e.g., probes with a frequency of at least 12 mHz and acceptable power Doppler sensitivity). All participating ultrasonographers were mandated to undertake study-specific training.

Scans will be performed at baseline and 6, 12, 18 and 24 months (and at any point between where the supervising physician believes that the study subject has achieved the primary endpoint). At each scanning visit, designated sonographers will scan a core set of joints including (but not confined to) dorsal views of the wrists, metacarpophalangeal (MCP 1–3), proximal interphalangeal (PIP 2, 3) and metatarsophalangeal (MTP 2–5) joints. Grading of grayscale and power Doppler measurements will be documented by applying semi-quantitative scales dictated by atlases provided to each participating centre. The scanning process will be scheduled before treatment is initiated for the baseline scan, and within 2 weeks either side of scheduled 6-, 12-, 18- and 24-month visits, if scans cannot be accommodated at the same time as scheduled visits.

In addition to the above assessments, ultrasound evaluation will be performed to confirm whether the study subject has met the primary outcome and will include the core set of joints, as above, as well as imaging of any additional symptomatic joints. Ultrasound assessments and scores will be added to the electronic case report form upon completion of the imaging study and scores will be blinded to the supervising PI or clinical assessor. Anonymised copies of images will be either stored on CDs and securely mailed or sent electronically to a secure email address in a central unit so that scores can be reviewed to ensure consistency of assessments across centres.

#### X-ray imaging

All x-ray images will be uploaded onto a dedicated, password-protected web-based system and will be scored centrally.

#### Routine clinical laboratory tests

Study subjects will undergo routine blood monitoring to screen for biological therapy-related toxicity at all assessments or in accordance with local practice if more frequent. Routine blood monitoring beyond the 12-month study visit will be taken only if clinically indicated and will be left to the discretion of the supervising physician.

#### Exploratory laboratory tests

These assays will be undertaken by the study investigators or designated collaborators either within or outside of the UK, as agreed and designated by the APIPPRA study investigators. Samples of blood and urine will be transported from recruiting sites across the UK and the Netherlands to pre-designated laboratories based in academic centres for processing and storage. Subsequently, all samples will be shipped from processing hubs in batches to the UK Biocentre for long-term storage prior to distribution to the relevant research laboratory for subsequent biomarker analysis as outlined below. The schedule of blood and urine sampling is summarised in Table 2.

**Table 2:** Schedule of blood and urine sampling

Biological Sample	Type & No.	Tube Cap Colour	Baseline	Weeks 13	Weeks 26	Weeks 39	Weeks 52	Weeks 65	Weeks 78	Weeks 91	Weeks 104
Serum	SST x 2	Red	18ml^a^	18ml	18ml	18ml	18ml	18ml	18ml	18ml	18ml
PBMC	Heparin x 5	Green	40ml	40ml	40ml	40ml	40ml	40ml	40ml	40ml	40ml
RNA	Tempus x 2	Blue	5ml	5ml	5ml	5ml	5ml	5ml	5ml	5ml	5ml
DNA	EDTA x 1	Purple	7ml								
Routine drug monitoring^b^	According to local procedures			10ml	10ml	10ml	10ml	10ml	10ml	10ml	10ml
Urine	Plain x 1	Beige	20ml	20ml	20ml	20ml	20ml	20ml	20ml	20ml	20ml
Total volume of blood draw			70ml	73ml	73ml	73ml	73ml	73ml	73ml	73ml	73ml

^a^reflects volume of whole blood draw into sample specific tubes

^b^up to 20 mls will be collected

### Study endpoints

#### Primary endpoint

The primary endpoint of this study is the time to development of clinical synovitis or RA defined by one of the following methods, whichever is met first:

1. The time to development of clinically apparent synovitis in at least three joints, as determined by two independent assessors with experience in clinical assessment of RA.
2. The time to development of RA according to the ACR/EULAR 2010 classification criteria [19], where joint involvement is defined as joint swelling. The ACR/EULAR 2010 classification criteria for RA redefines previous paradigms of RA by focusing on features at earlier stages of disease that best discriminate those features associated with persistent and/or erosive disease from those that do not. They were defined by consensus with the aim of identifying a disease state for which starting disease-modifying therapy was deemed appropriate.

**Fig. 2:**
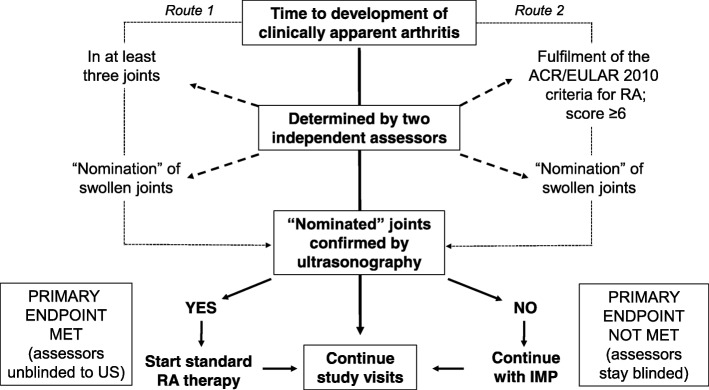
Primary endpoint roadmap

For either endpoint, joint swelling will be confirmed by ultrasonography (Fig. 2). If the primary endpoint is not confirmed, study participants continue IMP.

#### Secondary endpoints

The secondary endpoints of this study are the following:

1. The development of RA according to the ACR/EULAR 2010 criteria where joint involvement based on ultrasound assessments will be included.
2. Assessments of disease activity and progression over time, including the following:
  DAS28 (tender and swollen joint counts, patient global VAS, and ESR) and extended joint count 68/66
  SDAI and CDAI
  Pain VAS
  Lifestyle Factors Questionnaire, HAQ, Modified Illness Perception Questionnaire (Modified IPQ) and Euro-Quality of Life Questionnaire (EQ-5D)
  Hospital Anxiety and Depression Scale (HADS)
  RA–Work Instability Scale (RA-WIS)
  Functional Assessment of Chronic Illness Therapy–Fatigue Questionnaire (FACIT-F)
  Symptoms in Persons At Risk of Rheumatoid Arthritis (SPARRA) questionnaire
3. The proportion of participants requiring DMARD therapy and the time to commencing DMARD therapy, including oral or parenteral corticosteroids
4. Progression of radiographic changes in x-rays of the hands and feet scored by van der Heijde–Sharp Modified Scores or using the Larsen score
5. Changes in scores of synovitis and vascularity defined by ultrasonography and power Doppler over time
6. Adverse events
7. A Perceptions of Trial Participation Questionnaire was also included to gain insights into the acceptability of this therapeutic approach.

#### Exploratory endpoints

1. Changes in serum ACPA levels over time
2. ACPA isotype and antigenic fine specificity over time
3. Signatures of immune and inflammatory responses as defined through analysis of serum, peripheral blood cell subsets, peripheral blood RNA expression profiling and urine.

The focus of the proposed biomarker analysis is twofold. First, autoantibody profiles, immune cell phenotyping and gene expression signatures will be interrogated to identify novel signatures associated with a high-risk state. This could be used to stratify patients for future prevention studies. Second, we will use similar assays to better understand the mechanisms whereby study subjects respond to abatacept.

### Statistical considerations

#### Randomisation procedure, blinding and data management

If the participant is willing to participate in the trial, informed consent will be obtained at the start of the screening visit (Additional files 1 and 2). Screening assessment data will be entered by sites onto a web-based InferMed MACRO Electronic Data Capture (EDC) system hosted on a dedicated secure website by King’s College London, using only the participant’s initials and date of birth as identifiers (Additional file 3). The EDC system will automatically assign a unique participant identification number (PIN) to each participant as they are registered onto the EDC system. Participants who consent to screening but who are subsequently found to be ineligible will also be recorded in the EDC system for CONSORT (Consolidated Standards of Reporting Trials) reporting purposes. These procedures will operate in accordance with the International Conference on Harmonisation of Technical Requirements for Registration of Pharmaceuticals for Human Use guidelines for good clinical practice (GCP), meeting the requirements of the Medicines and Healthcare products Regulatory Agency.

Once participants are confirmed eligible, authorised study site staff will access the web-based randomisation system and enter the PIN, initials and date of birth of the participant along with details of any characteristics to be used in the randomisation algorithm. Staff at individual centres and the sponsor will be unaware of the allocation sequence.

Participants will be randomly assigned to IMP (abatacept) and placebo groups by using the method of stratified randomisation with randomly permuted blocks within strata defined by gender, country (UK and the Netherlands) and smoking status (never, previous and current). Once the participant is randomly assigned, the system will automatically recognise what active and placebo kit numbers are available in the site pharmacy and will select four blinded treatment kit numbers from the appropriate trial arm and this will be allocated to the participant. At each follow-up visit, site staff will again access the randomisation system and allocate new treatment kits for the participant. All participants and staff involved in the conduct of the study will be blind to treatment allocation throughout the trial.

#### Assessment of safety

All subjects who receive study drugs will be evaluated for safety. Safety outcomes include adverse events, clinically significant changes in vital signs, laboratory test abnormalities, and clinical tolerability of the drug. The investigator will determine the severity of each adverse event as mild, moderate, severe or very severe. Laboratory findings that the investigator feels are clinically relevant will be recorded as adverse events. In addition, the investigator will determine the relationship of the adverse event to the administration of the study drug. Any occurrence of a serious adverse event (SAE) from time of consent forward, up to and including follow-up visits, will be reported.

#### Sample size

Using time to development of arthritis as the primary outcome, or development of RA within 24 months of follow-up, according to 2010 ACR/EULAR criteria, a total of 52 study subjects need to present with this endpoint from 206 randomly assigned, based on the following information. It is conservatively anticipated that 40% of participants on placebo will develop arthritis. A sample size of 172 subjects would be needed to provide 80% power to detect a 50% relative reduction to 20% in the abatacept arm (a hazard ratio of 0.437) on the basis of a two-sided log-rank test at the 5% level of significance without loss to follow-up of any of the required 52 events. Therefore, 206 subjects (103 per arm) will be randomly assigned to allow for loss of events due to dropout, applying a conservative 20% inflation for dropout.

Similar effect sizes are detectable for binary outcomes and for other time-to-event and proportion outcomes such as the development of RA according to ACR/EULAR 2010 criteria alone. For continuous secondary outcomes, such as DAS measures, a medium effect size difference in means (of size 0.5 of a standard deviation, or SD) between the arms, based on the two-sided unpaired *t* test at the 5% significance level, can be detected with 85% to 90% power if 146 to 172 subjects are followed up. In view of the number of required secondary outcomes, with 172 participants followed up, there is also 80% power to detect medium-sized effects in secondary outcomes (0.52 of a SD) using a secondly applied significance level of 1%. Analyses incorporating baseline adjustment or repeated measures data (or both) will provide increased precision.

In view of the large number of secondary outcomes and the fact that the sample size was not set to detect powered effects specifically on the scale of these secondary measures, the use of a 1% level of significance goes only partway towards addressing the fact that multiple testing increases the chance that significant secondary findings are false. The results from secondary outcome analyses will be interpreted cautiously and in relation to the estimated confidence limits on the actual scale of the measures. Significance tests will be used sparingly and restricted where possible to address the stated hypotheses. Results that are significant in isolation will be interpreted less strongly than the set of results that are mutually supportive or that support the corresponding primary outcome or that are supported in previous research findings.

#### Withdrawal of subjects

Participants will be free to withdraw at any time. Participants who do withdraw from IMP will be invited to return for milestone assessments (at months 3, 6, 9 and 12 or at months 15, 18, 21 and 24, depending on the phase of the study) so that data may be collected and changes in their disease can be assessed; it will be made clear to them that this is entirely at their own discretion.

Subjects will discontinue investigational product (and non-investigational product at the discretion of the investigator) for any of the following reasons:

1. Withdrawal of informed consent (subject’s decision to withdraw for any reason) (Additional files 1 and 2).
2. Any clinical adverse event, laboratory abnormality, or intercurrent illness which, in the opinion of the PI, indicates that continued participation in the study is not in the best interest of the subject.
3. In the PI’s opinion, the need to administer concomitant medication not permitted by the trial protocol.
4. Pregnancy.

All subjects who discontinue should comply with protocol-specified follow-up procedures. The only exception to this requirement is when a subject withdraws consent for all study procedures or loses the ability to consent freely. If a subject withdraws before completing the study, the reason for withdrawal will be documented appropriately.

#### Statistical analysis

The primary analysis approach will be an intention-to-treat (ITT) strategy, including sensitivity analysis for missing data [20], sensitivity analysis for low compliance [21], and sensitivity analysis for use of forbidden rescue medication or potential informative dropout (described in the detailed statistical analysis plan).

For each time-to-event outcome, including the primary outcome, Kaplan–Meier survival curves will be estimated. A Cox stratified proportional hazards regression model, accounting for the randomisation stratifiers in the form of nominal categorical variables, will be used to compare randomised arms and obtain an estimated hazard ratio for the treatment effect with 95% confidence interval. Participant follow-up is on a three-monthly basis, and there will be additional monthly phone calls when participants are able to additionally report on their progress. Days will be the unit of time within the model in order to fully capture the inevitable variation in the time from baseline to monthly contacts and to ascertained outcome events. Each participant who drops out will be included in the analysis and will be assumed not to have the event and be censored at the point of dropout. Time will also be censored for those reaching the 24-month follow-up without an event. Those with an event will not contribute any further time after the event. For the primary outcome, sensitivity analysis will be carried out. This includes an analysis assuming that those who have dropped out for reasons connected with disease severity are alternatively deemed to have had the event and an analysis assuming this alternative for those having experienced two affected joints.

For each binary outcome, a stratified difference in proportions using the Cochran–Mantel–Haenszel method will be used to compare arms accounting for the randomisation stratifiers in their nominal categorical form. For each continuous outcome, including DAS28, differences in the mean of the outcome between arms will be estimated by using a linear mixed effects regression model of the repeated measures of the outcome across follow-up. ‘Visit’ will be included in the model as a continuous covariate with full polynomial terms equivalent to including ‘Visit’ as a categorical factor. Outcome data will be included from the regular visits and from times of measurement, including at ultrasound visits. The model will have a heterogeneous variance first-order autoregressive covariance structure, where measurements at the non-regular visits are included in the variance part of the model at the nearest visit and are included in the mean part of the model in proportion to how the measurement was timed between two visits. The ‘visit’ covariate will be located with an origin at 24 months, so that the model with the interactions of the ‘time’ terms with the other included main effect covariates of study arm, the randomisation stratifiers, and the baseline of the outcome and its missing indicator will directly provide the estimate of the study arm effect and its standard error. The missing indicator method [21] will be used to enable those participants having any outcome data, but with missing baseline data, to contribute to the estimate.

Descriptive statistics will be reported for measures of acceptability, feasibility and safety. Percentage measures will be reported with exact 95% confidence intervals. There is no plan to have stopping rules. It is anticipated that the data monitoring committee (DMC) will request interim data on safety and will advise on further data required for monitoring and on trial statistician blinding status. A detailed statistical analysis plan will be developed from the study protocol prior to the availability of follow-up data for approval by the independent trial steering committee (TSC). The primary analysis will follow the ITT principle; that is, participants will be analysed in the groups to which they were randomly assigned irrespective of treatment received, using all available follow-up data from all randomly assigned participants, with a per-protocol analysis of compliers only, as defined in the statistical analysis plan. Alterations to the statistical analysis plan will require re-approval from the TSC.

#### Trial oversight

An independent DMC will assess the trial’s progress, occurrence of adverse events and all other aspects. It will comprise a committee chair, the APIPPRA study trial statisticians, one independent statistician, and at least two independent members with experience in RA trials. The DMC will also be responsible for monitoring evidence of harm and for reviewing decisions relating to all aspects of safety reporting. They will meet prior to initiation of the study and at about 6 monthly intervals, or at more frequent intervals as deemed appropriate, for the duration of the study. The statistical analytical plan will be used to guide decision making.

The TSC was formed to provide oversight of this trial and ensure that it is being conducted in accordance with the principles of GCP and the relevant regulations. The TSC will approve the trial protocol and any protocol amendments and will provide advice to the investigators on all aspects of the study. The TSC will include an independent chair, the CI and core study team, an independent statistician, at least two clinicians with experience in RA trials who are not otherwise involved in the study, and an experienced patient expert as patient representative. The TSC is the main decision-making body and will be responsible for trial conduct and scientific direction and will ensure that the study objectives are achieved in a timely fashion and within budget.

### Data management

InferMed MACRO EDC will be used in this study. The King’s Clinical Trials Unit (KCTU) has extensive experience with this system. Password management and data exports will be controlled by the KCTU. No outcome data will be exported without the explicit consent of the trial statistician. Changes to the EDC system once the study has begun will be minimised and will be undertaken only with the full agreement of the trial statistician, CI and the KCTU where it is essential to the successful conduct of the study.

### Direct access to source data and documents

Site investigator(s) will permit trial-related monitoring, audits, and regulatory inspections (where appropriate) by providing the sponsors and regulators with direct access to source data and other documents (e.g., hospital case notes, electronic patient records, completed forms and questionnaires, and the investigator site file). All reasonable precautions to maintain the confidentiality of participants’ identities and protect the integrity of the data will be taken.

### Quality assurance

This trial will be monitored to ensure compliance with the trial protocol, GCP and all applicable regulations and to protect scientific integrity. Study management staff will undertake routine quality-control checks of the data. This will include additional central and site-based data checking to ensure that the data quality is accurate. Data queries will be raised, responded to and closed within the EDC system. Range and validation checks will be programmed into the EDC system to minimise transcription errors. Source data verification checks undertaken onsite will be documented to ensure that the final dataset has not been amended after checks have been completed. Checks of randomisation data will be undertaken periodically to identify any errors. Prior to database lock, all SAEs reported via fax or email will be cross-checked with the EDC system to ensure that all are present in the analysis dataset. Any data issues identified by the trial statistician during preparation of DMC reports will be reported to the trial coordinator and systematically rectified across the dataset, through either central or site data checks.

### Discussion

This study protocol describes a secondary prevention strategy for a common immune-mediated inflammatory disease targeting a phase of the disease process for which there is currently no recognised treatment. The trial will recruit one of the largest populations of “at risk” subjects to a randomised clinical trial described to date.

The APIPPRA study, designed by clinicians, experienced trialists and patients, is unusual in several respects. Rather than testing the acceptability and efficacy of an unlicensed IMP in a population of patients with established disease, this study will explore the effects of a licensed biologic DMARD in a population of otherwise healthy individuals deemed to be at risk of developing RA [22]. When the study was first conceived, the phenotype of at-risk subjects was only beginning to emerge. In 2012, a EULAR working group published recommendations describing the natural history of RA, highlighting how each phase of the disease corresponded to distinct clinical and laboratory characteristics [23]. These recommendations were informed in part by longitudinal observational studies of at-risk subjects. These and many other studies have been pivotal to stratifying those at highest risk [22, 24–27], in whom therapeutic intervention is considered to be appropriate, and have provided a framework for estimating progression rates and for computing sample size calculations for our study.

The armamentarium for treating established RA has grown substantially over the last two decades, and this, together with intensive, target-driven treatment strategies [1, 5, 28], has had a major impact on disease outcomes; remission rates have approached 40% within 6 months of commencing therapy. There is growing appreciation, however, that the efficacy of interventions may depend on the specific phase of the disease (reviewed in [29]). For example, it remains to be determined whether targeting inflammatory cytokines during the pre-clinical phase of RA at a time when the inflammatory burden of disease is minimal would be as beneficial as immunomodulatory drugs. Clinical trials of corticosteroids, for example, have not demonstrated durable clinical outcomes in the at-risk population [30–32]. Co-stimulatory blockade with abatacept targets one of the earliest phases of the disease process—attenuating *de novo* activation of self-reactive T cells by antigen-presenting cells. Abatacept has proven efficacy in early and established RA, but the drug has never been tested in subjects prior to the onset of clinically apparent arthritis. Nonetheless, the presence of disease-associated autoantibodies in serum indicates that the autoimmune process has already started and so we hypothesise that interrupting these immune reactions with abatacept is a biologically plausible approach. Furthermore, there are currently no other licensed therapies available with a comparable safety profile that target adaptive immunity. Finally, the duration of IMP exposure used in the APIPPRA study is largely empirical. Clinical trials of abatacept in patients with recent-onset type I diabetes, however, have documented durable outcomes beyond the period of therapy, suggesting that immune modulation might be more profound, or sustained, if used at the earliest detectable point in the disease course [33]. Whether co-stimulatory blockade induces immunological tolerance, on the other hand, requires further investigation.

The primary endpoint of the APIPPRA trial is the time to the development of clinically swollen joints or fulfilment of the 2010 ACR/EULAR classification criteria for RA, whichever endpoint is met first. Three swollen joints were chosen for the first primary outcome since this was the median number of swollen joints identified from cohorts of at-risk subjects at the time of development of clinically apparent arthritis [9]. In routine practice, this is very often the point in the disease course when physicians see patients with inflammatory arthritis for the first time. The validity of the 2010 ACR/EULAR classification criteria for RA is underpinned by an intention to treat with disease-modifying drugs [19] and so including these criteria as a co-primary outcome (by achieving a score of at least 6) reduces the risk of exposing study subjects to placebo, or to IMP that has proven inadequate to suppress signs and symptoms, at a time when standard therapy for new-onset RA would be deemed appropriate by the supervising physician. Clinical assessments are notoriously subjective, especially when inflammatory joint disease is in the very earliest stages. To define primary endpoints with precision, we opted to confirm the presence of synovitis in clinically swollen joints by ultrasonography. For these imaging assessments and for the duration of the study, clinical assessors and ultrasonographers are blinded to each other’s joint scores to limit bias. For consistency of scoring, all ultrasonographers undergo study-specific training and have access to a reference atlas of images for all joints to be assessed.

A key outcome of the APIPPRA study will be to determine whether intervention at this phase of the disease is considered to be acceptable to the high-risk subject. To this end, we have included as part of study assessments questionnaires that probe in more detail people’s perception of risk. We anticipate that the APIPPRA study will allow us to better define the at-risk state on the basis of data we acquire from questionnaires that probe deeper into clinical phenotypes, from the monitoring of images of symptomatic joints by ultrasound over time, and from immune phenotypes acquired from analysis of biological samples. This information then can be exploited in future studies to identify those most likely to progress over pre-defined time periods, on the one hand, while minimising exposure of individuals to an intervention they may never need.

## Trial status

The APIPPRA study trial received ethical approval on 13 March 2014. The first study subject was randomly assigned in January 2015, and the trial completed recruitment at end of December 2018 (Additional file 3).

### Protocol version

The protocol published here is version 3.2, dated 22 March 2018.

## Additional files

Appendices 1: Informed consent form. (PDF 111 kb)

Appendices 2: Informed consent form for additional blood. (PDF 102 kb)

Appendices 3: Additional information. (DOCX 23 kb)

Additional file 4: SPIRIT (Standard Protocol Items: Recommendations for Interventional Trials) 2013 Checklist: Recommended items to address in a clinical trial protocol and related documents. (DOCX 54 kb)

## Data Availability

Further information regarding the logistics of RA prevention study operations is available from the authors upon reasonable request.

## Abbreviations

ACPA: Anti-citrullinated peptide antibody
ACR: American College of Rheumatology
APIPPRA: Arthritis Prevention In the Pre-clinical Phase of RA with Abatacept
CDAI: Clinical disease activity index
CI: Chief investigator
DAS: Disease activity score
DMARD: Disease-modifying anti-rheumatic drug
DMC: Data monitoring committee
EDC: Electronic data capture
ESR: Erythrocyte sedimentation rate
EULAR: European League Against Rheumatism
GCP: Good clinical practice
GP: General practitioner
HAQ: Health Assessment Questionnaire
IMP: Investigational medicinal product
ITT: Intention-to-treat
IV: Intravenous
KCTU: King’s Clinical Trials Unit
PI: Principal investigator
PIN: Participant identification number
RA: Rheumatoid arthritis
RF: Rheumatoid factor
SAE: Serious adverse event
SC: Subcutaneous
SD: Standard deviation
SDAI: Simple disease activity index
TB: Tuberculosis
TSC: Trial steering committee
ULN: Upper limit of normal
VAS: Visual analogue scale

## References

[1] Scott DL, Wolfe F, Huizinga TWJ (2010). Rheumatoid arthritis. Lancet.

[2] Hochberg MC, Spector TD (1995). Epidemiology of rheumatoid arthritis: update. Baillieres Clin Rheumatol.

[3] Wolfe F, Hawley DJ (1998). The longterm outcomes of rheumatoid arthritis: Work disability: a prospective 18 year study of 823 patients. J Rheumatol.

[4] Michaud K, Messer J, Choi HK, Wolfe F (2003). Direct medical costs and their predictors in patients with rheumatoid arthritis: a three-year study of 7,527 patients. Arthritis Rheum.

[5] Goekoop-Ruiterman YP, de Vries-Bouwstra JK, Allaart CF, van Zeben D, Kerstens PJ, Hazes JM (2005). Clinical and radiographic outcomes of four different treatment strategies in patients with early rheumatoid arthritis (the BeSt study): a randomized, controlled trial. Arthritis Rheum.

[6] Nielen MM, van Schaardenburg D, Reesink HW, van de Stadt RJ, van der Horst-Bruinsma IE, de Koning MH (2004). Specific autoantibodies precede the symptoms of rheumatoid arthritis: a study of serial measurements in blood donors. Arthritis Rheum.

[7] Rantapää-Dahlqvist S, de Jong BA, Berglin E, Hallmans G, Wadell G, Stenlund H (2003). Antibodies against cyclic citrullinated peptide and IgA rheumatoid factor predict the development of rheumatoid arthritis. Arthritis Rheum.

[8] Ioan-Facsinay A, Willemze A, Robinson DB, Peschken CA, Markland J, van der Woude D (2008). Marked differences in fine specificity and isotype usage of the anti-citrullinated protein antibody in health and disease. Arthritis Rheum.

[9] Bos WH, Wolbink GJ, Boers M, Tijhuis GJ, de Vries N, van der Horst-Bruinsma IE (2010). Arthritis development in patients with arthralgia is strongly associated with anti-citrullinated protein antibody status: a prospective cohort study. Ann Rheum Dis.

[10] Azuma M, Ito D, Yagita H (1993). B70 antigen is a second ligand for CTLA-4 and CD28. Nature (London).

[11] Freeman GJ, Gribben JG, Boussiotis VA (1993). Cloning of B7-2: a CTLA-4 counter receptor that costimulates human T cell proliferation. Science.

[12] Kremer JM, Westhovens R, Leon M, Di Giorgio E, Alten R, Steinfeld S (2003). Treatment of rheumatoid arthritis by selective inhibition of T-cell activation with fusion protein CTLA4Ig. N Engl J Med.

[13] Kremer JM, Dougados M, Emery P, Durez P, Sibilia J, Shergy W (2005). Treatment of rheumatoid arthritis with the selective costimulation modulator abatacept: twelve-month results of a phase iib, double-blind, randomized, placebo-controlled trial. Arthritis Rheum.

[14] Westhovens R, Robles M, Ximenes AC, Nayiager S, Wollenhaupt J, Durez P (2009). Clinical efficacy and safety of abatacept in methotrexate-naive patients with early rheumatoid arthritis and poor prognostic factors. Ann Rheum Dis.

[15] Genovese MC, Schiff M, Luggen M, Becker JC, Aranda R, Teng J (2008). Efficacy and safety of the selective co-stimulation modulator abatacept following 2 years of treatment in patients with rheumatoid arthritis and an inadequate response to anti-tumour necrosis factor therapy. Ann Rheum Dis.

[16] Emery P, Durez P, Dougados M, Legerton CW, Becker JC, Vratsanos G (2010). Impact of T-cell costimulation modulation in patients with undifferentiated inflammatory arthritis or very early rheumatoid arthritis: a clinical and imaging study of abatacept (the ADJUST trial). Ann Rheum Dis.

[17] Genovese MC, Covarrubias A, Leon G, Mysler E, Keiserman M, Valente R (2011). Subcutaneous abatacept versus intravenous abatacept: a phase IIIb noninferiority study in patients with an inadequate response to methotrexate. Arthritis Rheum.

[18] Felson DT, Smolen JS, Wells G, Zhang B, van Tuyl LH, Funovits J (2011). American College of Rheumatology/European League against Rheumatism provisional definition of remission in rheumatoid arthritis for clinical trials. Ann Rheum Dis.

[19] Aletaha D, Neogi T, Silman AJ, Funovits J, Felson DT, Bingham CO (2010). 2010 Rheumatoid arthritis classification criteria: an American College of Rheumatology/European League Against Rheumatism collaborative initiative. Arthritis Rheum..

[20] White IR, Horton NJ, Carpenter J, Pocock SJ (2011). Strategy for intention to treat analysis in randomised trials with missing outcome data. BMJ.

[21] White IR, Kalaitzakib E, Thompson SG (2011). Allowing for missing outcome data and incomplete uptake of interventions, with application to an internet-based alcohol trial. Stat Med.

[22] van Steenbergen HW, Aletaha D, de Beaart-van (2017). EULAR definition of arthralgia suspicious for progression to rheumatoid arthritis. Ann Rheum Dis.

[23] Gerlag DM, Raza K, van Baarsen EGM, Brouwer E, Buckley CD, Burmester GR (2012). EULAR recommendations for terminology and research in individuals at risk of rheumatoid arthritis (RA): Report from the Study Group for Risk Factors for RA. Ann Rheum Dis.

[24] van der Woude D, Rantapaa-Dahlqvist S, Ioan-Facsinay A (2010). Epitope spreading of the anti-citrullinated protein antibody response occurs before disease onset and is associated with the disease course of early arthritis. Ann Rheum Dis.

[25] Nam JL, Hensor EM, Hunt L, Conaghan PG, Wakefield RJ, Emery P (2016). Ultrasound findings predict progression to inflammatory arthritis in anti-CCP antibody-positive patients without clinical synovitis. Ann Rheum Dis.

[26] van de Stadt LA, Bos WH, Meursinge Reynders M (2010). The value of ultrasonography in predicting arthritis in autoantibody positive arthralgia patients: a prospective cohort study. Arthritis Res Ther.

[27] van Steenbergen HW, Mangnus L, Reijnierse M, Huizinga TW, van der Helm-van Mil AH (2016). Clinical factors, anticitrullinated peptide antibodies and MRI-detected subclinical inflammation in relation to progression from clinically suspect arthralgia to arthritis. Ann Rheum Dis..

[28] Aletaha D, Smolen JS (2018). Diagnosis and Management of rheumatoid arthritis: a review. JAMA.

[29] Cope AP (2017). Emerging therapies for pre-RA. Best Pract Res Clin Rheumatol.

[30] van Dongen H, van Aken J, Lard LR, Visser K, Ronday HK, Hulsmans HM (2007). Efficacy of methotrexate treatment in patients with probable rheumatoid arthritis: a double-blind, randomized, placebo-controlled trial. Arthritis Rheum.

[31] Verstappen SM, McCoy MJ, Roberts C, Dale NE, Hassell AB, Symmons DP, STIVEA investigators (2010). Beneficial effects of a 3-week course of intramuscular glucocorticoid injections in patients with very early inflammatory polyarthritis: results of the STIVEA trial. Ann Rheum Dis.

[32] Bos WH, Dijkmans BA, Boers M, van de Stadt RJ, van Schaardenburg D (2010). Effect of dexamethasone on autoantibody levels and arthritis development in patients with arthralgia: a randomised trial. Ann Rheum Dis.

[33] Orban T, Bundy B, Becker DJ, DiMeglio LA, Gitelman SE, Goland R (2011). Co-stimulation modulation with abatacept in patients with recent-onset type 1 diabetes: a randomised, double-blind, placebo-controlled trial. Lancet.

