# Supplementary material for: Arthritis prevention in the pre-clinical phase of RA with abatacept (the APIPPRA study): a multi-centre, randomised, double-blind, parallel-group, placebo-controlled clinical trial protocol
Source: Trials. 2019 Jul 15;20:429. doi: 10.1186/s13063-019-3403-7 (PMC6633323; doi:10.1186/s13063-019-3403-7)
Supplement: Supplementary file 1 — Informed consent form. (PDF 111 kb) [file 13063_2019_3403_MOESM1_ESM.pdf]

APIPPRA

Arthritis Prevention
In The Pre-Clinical Phase Of RA
With Abatacept

*To be printed on Local Trust Headed Paper*

| | |
|---|---|
| **Centre Name:** | |
| **Participant Identification Number (PIN):** | |
| **Name of Principal Investigator:** | |

# THE APIPPRA STUDY

### Arthritis Prevention In the Pre-clinical Phase of Rheumatoid Arthritis with Abatacept

## CONSENT TO PARTICIPATE IN THE APIPPRA STUDY

| | Please Initial Boxes |
|---|---|
| 1. I confirm that I have read and fully understand the Participant Information Sheet Version 2.2, dated 20/03/2018, for the APIPPRA study. | ☐ |
| 2. I have had the opportunity to consider the study, ask questions and have had these answered satisfactorily. | ☐ |
| 3. I understand that my participation in this study is voluntary and that I may withdraw from the study at any time without giving a reason and without my routine medical care or legal rights being affected. | ☐ |
| 4. I agree to donate blood and urine samples at each assessment and understand that these samples will be used for genetic and biomarker research into the development of rheumatoid arthritis (RA), as outlined in the Participant Information Sheet. | ☐ |
| 5. I agree to the storage of my blood and urine samples in a Biobank for further research studies into the development of RA and understand that these studies have been approved by a Research Ethics Committee. | ☐ |
| 6. I understand that biomarker analysis on the samples I provide will be undertaken by the APIPPRA study investigators and their designated collaborators (including industry partners) either within or outside the UK, and that this analysis will involve the use of anonymised medical information collected during the study. | ☐ |
| 7. I understand that although all information collected about me during the course of the research will be kept strictly confidential, access to relevant sections of my medical notes and data collected during the study will be provided to staff from the organisation who are coordinating and are responsible for the study, regulatory authorities and the clinical study team, where it is relevant to my taking part in this research. I understand that anonymised information from the study may also be shared with the company that produces the drug (Bristol-Myers Squibb). I give permission for these individuals to have access to my records. | ☐ |

8. I understand that I do not own or have any rights to the samples or information that comes from the sample analysis, and that I will not benefit financially if the research leads to the development of a new treatment or medical test. ☐

9. I agree to my GP being informed of my participation in the study. ☐

10. I agree to take part in the APIPPRA study. ☐

11. I agree to be contacted in the future to provide information about participation in clinical trials and research studies. ☐

| ______________________ | ______________________ | ______________________ |
| --- | --- | --- |
| **Name of Participant** | **Signature** | **Date** |
| | | |
| **Name of Person taking consent** | **Signature** | **Date** |

**CONSENT TO COMPLETE PERCEPTIONS OF TRIAL PARTICIPATION QUESTIONNAIRE ONLY**

**Please Initial Boxes**

1. I have chosen not to participate in the APIPPRA study ☐

2. I agree to complete a questionnaire about my reasons for not taking part in the study (as well as information about my lifestyle and wellbeing), which will help with the design of future research studies. I understand that the information I provide will be kept strictly confidential. ☐

3. I agree to be contacted in the future to provide information about participation in clinical trials and research studies. ☐

| ______________________ | ______________________ | ______________________ |
| --- | --- | --- |
| **Name of Participant** | **Signature** | **Date** |
| | | |
| **Name of Person taking consent** | **Signature** | **Date** |

***Please place original signed consent form in the Investigator site file, and provide one copy for the medical notes, and a second copy for the participant***

**This study is supported by the NIHR Translational Research Partnerships and the NIHR Clinical Research Network**
