# Supplementary material for: Arthritis prevention in the pre-clinical phase of RA with abatacept (the APIPPRA study): a multi-centre, randomised, double-blind, parallel-group, placebo-controlled clinical trial protocol
Source: Trials. 2019 Jul 15;20:429. doi: 10.1186/s13063-019-3403-7 (PMC6633323; doi:10.1186/s13063-019-3403-7)
Supplement: Supplementary file 2 — Informed consent form for additional blood. (PDF 102 kb) [file 13063_2019_3403_MOESM2_ESM.pdf]

APIPPRA

Arthritis Prevention
In The Pre-Clinical Phase Of RA
With Abatacept

To be printed on Local Trust Headed Paper

**Centre Name:**	
**Participant Identification Number (PIN):**	
**Name of Principal Investigator:**	

THE APIPPRA STUDY

Arthritis Prevention In the Pre-clinical Phase of Rheumatoid Arthritis with Abatacept

CONSENT FORM FOR ADDITIONAL BLOOD SAMPLE

Please Initial Boxes

(in the event of loss of sample)

1. I agree to donate extra blood and urine samples due to the unavoidable loss of previously taken samples and understand that these samples will be used for genetic and biomarker research into the development of rheumatoid arthritis (RA), as outlined in the Participant Information Sheet Version 2.2, dated 20/03/2018. ☐
2. I agree to the storage of these extra blood and urine samples in a Biobank, for further research studies into the development of RA and understand that these studies have been approved by a Research Ethics Committee. ☐
3. I understand that biomarker analysis on the samples I provide will be undertaken by the APIPPRA study investigators and their designated collaborators (including industry partners) either within or outside the UK, and that this analysis will involve the use of anonymised medical information collected during the study. ☐
4. I understand that I do not own or have any rights to the samples or information that comes from the sample analysis, and that I will not benefit financially if the research leads to the development of a new treatment or medical test. ☐

**Name of Participant**	**Signature**	**Date**
**Name of Person taking consent**	**Signature**	**Date**

Please place original signed consent form in the Investigator site file, and provide one copy for the medical notes, and a second copy for the participant

KING'S College LONDON

Guy's and St Thomas' NHS
NHS Foundation Trust

UKCRC Registered Clinical Trials Units

This study is supported by the NIHR Translational Research Partnerships and the NIHR Clinical Research Network
