# Supplementary material for: Arthritis prevention in the pre-clinical phase of RA with abatacept (the APIPPRA study): a multi-centre, randomised, double-blind, parallel-group, placebo-controlled clinical trial protocol
Source: Trials. 2019 Jul 15;20:429. doi: 10.1186/s13063-019-3403-7 (PMC6633323; doi:10.1186/s13063-019-3403-7)
Supplement: Supplementary file 3 — Additional information. (DOCX 23 kb) [file 13063_2019_3403_MOESM3_ESM.docx]

**APPENDIX 3: ADDITIONAL INFORMATION.**

**Short Title**

APIPPRA Trial

**Trial Registration ISRCTN**

ISRCTN46017566 REC Number: 14/LO/0100

**Trial Sponsors**

The APIPPRA study is co-sponsored by

King’s College London

Address: Strand, London, WC2R 2LS

Guys and St Thomas’ NHS Foundation Trust

16th floor, Tower Wing, Guy's Hospital

Town/city: London

Post code: SE1 9RT

Telephone: 020 7188 7188 ext: 54426

Fax: 020 7188 3472

E-mail: [R&D@gstt.nhs.uk](mailto:R&D@gstt.nhs.uk)

**Chief Investigator**

*Name*: Professor Andrew P Cope

Professor of Rheumatology

*Address*

Centre for Inflammation Biology and Cancer Immunology

School of Immunology and Microbial Sciences

Faculty of Life Sciences & Medicine

King’s College London

1st Floor, New Hunt's House

Great Maze Pond, London SE1 1UL

Tel: 0207 848 6901

Fax: 0207 848 8632

Email: [andrew.cope@kcl.ac.uk](mailto:andrew.cope@kcl.ac.uk)

**Trial Statistician.**

**Professor Toby Prevost**

Imperial Clinical Trials Unit, School of Public Health, Imperial College London, Stadium House, 68 Wood Lane, London W12 7RH.

Tel: 020 7594 8805

Email: [a.prevost@imperial.ac.uk](mailto:a.prevost@imperial.ac.uk)

**Trial Coordinating Team**

Clinical Trials Group, School of Immunology and Microbial Sciences, Faculty of Life Sciences and Medicine, King’s College London, Weston Education Centre, Cutcombe Road, Denmark Hill, London SE5 9RJ.

Tel: +44(0)20 7848 0852

Fax: +44(0)20 7848 5202

[apippra@kcl.ac.uk](mailto:apippra@kcl.ac.uk)

**Recruiting study sites**

***The UK sites***

1. Guy's & St Thomas's NHS Foundation Trust
2. King's College Hospital NHS Foundation Trust
3. University College London Hospitals NHS Trust
4. Basildon and Thurrock University Hospitals NHS Foundation Trust
5. Cambridge University Hospital NHS Foundation Trust
6. Glasgow Royal Infirmary
7. Newcastle upon Tyne NHS Foundation Trust
8. Great Western Hospitals NHS Foundation Trust
9. Oxford University Hospitals NHS Foundation Trust
10. The Dudley Group of Hospitals NHS Foundation Trust
11. New Queen Elizabeth Hospital UHB & City (Birmingham)
12. Birmingham City Hospital
13. Cannock Chase Hospital, The Royal Wolverhampton NHS Trust
14. Haywood Hospital, Midlands Partnership NHS Foundation Trust
15. The Leeds Teaching Hospital NHS Trust
16. Central Manchester University Hospitals NHS Foundation Trust
17. Nottingham University Hospitals NHS Trust
18. Royal Berkshire NHS Foundation Trust
19. Countess of Chester Hospital NHS Foundation Trust
20. Maidstone & Tunbridge Wells NHS Trust
21. New Cross Hospital, The Royal Wolverhampton NHS Trust
22. Homerton University Hospital NHS Foundation Trust
23. Luton & Dunstable Hospital NHS Foundation Trust
24. Heart of England NHS Foundation Trust
25. Ashford and St Peter's Hospitals NHS Foundation Trust
26. Northampton General Hospital NHS Trust
27. Hull Royal Infirmary
28. University Hospitals Coventry and Warwickshire NHS Trust

***The Netherlands Sites***

1. Leiden University Medical Center
2. Atrium MC / Zuyderland
3. Reade

**Recruitment**

Patients will be recruited from 31 centres in UK and the Netherlands who are attending early rheumatology clinics. The numbers of centres and the duration of recruitment will be extended as needed if patient recruitment is slower than anticipated.

**Ethics and Regulatory Approvals**

The APIPPRA trial will be conducted in compliance with the principles of the Declaration of Helsinki (1996), the principles of GCP and in accordance with all applicable regulatory requirements including but not limited to the Research Governance Framework. This protocol and related documents will be submitted for review to a Research Ethics Committee (REC) and The Medicines and Healthcare Products Regulatory Agency (MHRA). Any amendments to approved documents or newly created documents will be submitted for approval.

# Annual Progress Report (APR) and Development Safety Update Report (DSUR) and a final report at conclusion of the study will be submitted to the REC and MHRA within the timelines defined in the Regulations.

**Data Handling**

The Chief Investigator will be the custodian of the trial data once the study is complete.

The following guidelines will be strictly adhered to:

- Data will be entered on the EDC system (InferMed MACRO), hosted by the King’s Clinical Trial Unit (KCTU) and stored on a KCL server
- The eCRF/EDC will be accessible via web-based secure application. Usernames and passwords will be used to authenticate each individual user. The trial manager will request access for relevant personnel and all access must be authorised by the trial manager. Different roles may be assigned, a data entry role allows site staff to enter data and respond to data discrepancies. A monitor role allows co-ordinating centre staff or sponsor monitors to review data, raise and close discrepancies, source data verify data items but not amend any data.
- Only designated, suitably trained clinical and designated research staff will be given access to the data. The trial coordinator will only authorise passwords to personnel designated on the study’s signature and delegation log as requiring login details to enter data. Central password lists will not be stored external to the system.
- All study data will be stored and archived for an appropriate period of time in accordance with the Research Governance Framework for Health and Social Care (2005).

**Publication Policy**

It is intended that the results of the study will be reported and disseminated at international conferences and in peer-reviewed scientific journals. The Trial Steering Committee, together with the Chief Investigator and Principal Investigators will ensure that on completion of the study, the results are analysed, written up, reported and disseminated. Trial findings will be submitted to peer-reviewed journals, irrespective of the results of the study. Findings will also be publicised to the National Commissioning Board, other commissioners and appropriate established networks. And the outcomes of the trial will be disseminated to appropriate patient groups through national patient organisations. No professional writers will contribute to the trial outputs.

**Financial Aspects**

As this is a non-commercial research study, it is eligible for adoption onto the National Institute for Health Research (NIHR) Clinical Research Network Portfolio database, NHS Support Costs, including the additional patient-related costs associated with the research.

**Insurance and Indemnity**

Trial participants and staff working on the trial will be fully insured. Negligence will be covered by the participating NHS Trust's insurance. King's College London indemnity will cover non-negligent harm including that arising from the design of the research for both the UK and the Netherlands.
